# Knowledge, Attitude, and Practice (KAP) of ICU Nurses towards Tracheal Intubation Patients' Postextubation Dysphagia: A Cross-Sectional Study

**DOI:** 10.1155/2024/9981458

**Published:** 2024-05-31

**Authors:** Shanshan Sun, Lei Tao, Lin Yang, Zhigang Zhang

**Affiliations:** ^1^Department of Critical Care Medicine, The First Hospital of Lanzhou University, School of Nursing, Lanzhou University, Lanzhou, Gansu 730000, China; ^2^Sias University, Zhengzhou, Henan 450000, China

## Abstract

**Aim:**

The aim of this study was to understand the current status of knowledge-attitude-practice of ICU nurses in tertiary care hospitals regarding swallowing disorders after extubation of tracheally intubated patients and to analyse the influencing factors.

**Design:**

A cross-sectional study.

**Background:**

Most patients admitted to the ICU have an endotracheal tube, which may be the cause of acute and/or chronic problems after extubation. Therefore, training of ICU nurses and early extubation are essential to prevent these problems.

**Methods:**

A convenience sample of clinical nurses (*n* = 627) was selected from Grade A Hospitals in Northwest, North, and Central China as the study population. Survey instruments included the Questionnaire on ICU Nurses' Knowledge, Attitude, and Practice of Postextubation Swallowing Disorders in Patients with Tracheal Intubation. *Data Sources*. Data were sourced from structured questionnaire responses.

**Results:**

A total of 647 ICU nurses participated in this survey, with 627 valid questionnaires. The three dimensions of knowledge, attitude, practice, and the total score of the questionnaire on ICU nurses' knowledge, attitude, and practice of postextubation swallowing disorders in patients with tracheal intubation were (6.46±3.09), (7.53±1.69), (4.89±2.00), and (18.88±5.18), respectively. Multiple linear regression analysis showed that the factors affecting the total score of PED among ICU nurses were age, nationality, professional title, job satisfaction, and mode of employment. Gender, age, nationality, and job satisfaction were the factors that influenced the score of knowledge. The influencing factors of attitude score include gender, age, nationality, section, professional title, job satisfaction, and mode of employment. The influencing factors of the score of knowledge include professional title and job satisfaction.

**Conclusion:**

The current status of ICU nurses' knowledge-attitude-practice regarding postextubation dysphagia in tracheally intubated patients is generally at a moderate to low level, and the level of knowledge-attitude-practice needs to be further improved. *Implications for Nursing Management*. The results of the study showed that the knowledge, attitude, and practice of ICU nurses towards tracheal intubation patients' postextubation dysphagia were in the lower middle level. Therefore, it is necessary to improve the knowledge, attitude, and practice of ICU nurses towards tracheal intubation patients' postextubation dysphagia. This may include, but is not limited to, the development of tools for assessing PED, systematic and professional training, and the development of multidisciplinary collaborative models.

## 1. Introduction

Tracheal intubation is the most commonly used route for establishing an artificial airway and is mainly used for resuscitation and respiratory support in critically ill patients in the Intensive Care Unit (ICU) [1]. Patients with tracheal intubation are a special group of patients with postextubation dysphagia (PED) due to the potential for anatomical damage, which can cause oral, pharyngeal, and laryngeal lesions affecting local dynamics and sensitivity, impairing the swallowing protection response and leading to swallowing disorders [2–5]. A current foreign systematic evaluation found a 41.0% incidence of PED in adults with severe disease [6]. Other studies investigating PEDs in different disease types have shown that the incidence of PEDs ranges from 12.4% to 93.0% [2, 7–10], while studies in China have shown that the incidence of PEDs ranges from 7.1% to 67.2% [11–15]. In addition to having a high incidence, PEDs can have many adverse outcomes. Studies have shown that PED leads to increased risk of aspiration, aspiration pneumonia, delayed oral intake, malnutrition, prolonged ICU stay and total length of stay, increased mortality, etc. [16–18]. PED can persist until discharge and affect prognosis, even taking more than 6 months or years to recover, seriously affecting the efficiency of recovery [7, 19] and increasing the economic burden on the public health system [16].

In the clinical setting, ICU nurses are responsible for observing patients and providing daily care, and their knowledge, attitude, and practice towards PEDs can influence the occurrence and prognosis of PEDs. Studies have shown that ICU nurses can play an important role in managing PEDs and reducing adverse outcomes associated with swallowing [20–22]. Therefore, improving ICU nurses' knowledge, attitude, and practice about PEDs may help to improve the management of PEDs, reduce the impact of risk factors and avoid adverse outcomes, and promote patient recovery [23].

## 2. Background

Multidisciplinary teams of nurses, doctors, therapists, and speeching-language pathologists (SLPs) are emerging worldwide with effective screening and management processes. Surveys have shown that only 41% of hospitals routinely screen for PEDs and 44% of patients complete a swallowing assessment [24, 25], and less than 50% of hospitals in the USA have developed screening methods for PEDs, with rates of PED assessment varying between hospitals [26]. A review of the literature found that existing studies on the assessment of PEDs have been underemphasised and that there is a lack of relevant research and guideline norms [27].

In China and abroad, most studies related to PED have focused on investigating its incidence, influencing factors, or exploring the adverse outcomes caused by PED, but no studies have been conducted on ICU nurses' knowledge-attitude-practice regarding PED in tracheally intubated patients. In order to understand the awareness and importance of PEDs among ICU nurses, it is necessary to investigate the current status of their knowledge, attitude, and practice regarding PEDs in tracheally intubated patients and to explore the factors that influence knowledge, attitude, and practice. This will provide a basis for developing targeted training that will help to reduce the incidence of PEDs and adverse outcomes.

## 3. Methods

### 3.1. Aim

The aim of this study was to understand the current status of knowledge-attitude-practice of ICU nurses in tertiary care hospitals regarding swallowing disorders after extubation of tracheally intubated patients and to analyse the influencing factors.

### 3.2. Study Design

A cross-sectional survey design was used.

### 3.3. Sample and Setting

A convenience sampling method was adopted to select ICU nurses from selected Grade A Hospitals in Northwest, North, and Central China as the study population. The types of ICUs included general ICU and specialized ICU. Inclusion criteria were as follows: (1) those who obtained the nursing practice certificate; (2) ICU staff in service; and (3) informed consent and willingness to cooperate with the survey. Exclusion criteria were as follows: ICU nurses on rotation, refresher course, or internship. Based on the descriptive study sample size, the estimated sample size was calculated as 10–15 times the total number of entries in the questionnaire [28] and increased by 10% to eliminate the effect of the presence of unqualified questionnaires [29], as the finalised questionnaire had a total of 30 entries, i.e., sample size = number of entries × (10–15) × (1 + 10%) = (385–495), and finally the above sample size requirements and the composition of ICU nurses in the surveyed hospitals were integrated. Finally, 627 participants were enrolled in the study.

### 3.4. Instruments

The Questionnaire on ICU Nurses' Knowledge, Attitude, and Practice of Postextubation Swallowing Disorders in Patients with Tracheal Intubation consists of two parts: (i) a questionnaire on general demographic information of ICU nurses; and (ii) a questionnaire on ICU nurses' knowledge, attitude, and practice of postextubation swallowing disorders in patients with tracheal intubation (final questionnaire determined after item analysis and reliability testing), with a total of 30 items. Instrument details are given in supplemental files (available here).

#### 3.4.1. A Questionnaire on General Demographic Information of ICU Nurses

The demographic survey items measured gender, age, nationality, marital status, education, section, years of experience, title, whether they are a manager, satisfaction with their job, mode of employment, and frequency of night shifts.

#### 3.4.2. The ICU Nurses' Knowledge-Attitude-Practice Questionnaire on Postextubation Dysphagia in Patients with Tracheal Intubation

The questionnaire was developed through the construction of an initial pool of questionnaire items, two rounds of Delphi expert correspondence, item analysis, and reliability testing. A total of 300 questionnaires were sent out as reliability and validity tests, and 276 valid questionnaires were finally collected, with a recovery rate of 92.00%. The questionnaire consisted of 30 items in 3 dimensions: knowledge, attitude, and practice. Cronbach's alpha coefficient was 0.901, and the retest reliability was 0.990. The content validity (I-CVI) of each item ranged from 0.88 to 1.00 and the S-CVI/UA was 0.90. No exploratory factor analysis was required, and one common factor was extracted by exploratory factor analysis for the attitude and practice sections, with a cumulative variance contribution of 67.86% and 81.04%, which met the corresponding requirements.

### 3.5. Data Collection

With the help of the supervisor and relevant staff, the ICU nurse manager and the person in charge of the hospital being surveyed were contacted in advance to explain the content, purpose, and significance of the survey and to obtain cooperation. The survey was conducted using a uniform questionnaire guideline and method of completion and was carried out by the subject team members through paper or online questionnaires. The data were collected in Chinese and translated by the team. Then the English teacher of Lanzhou University was asked to proofread and finally translate the same into English.

### 3.6. Data Analysis

The data obtained from the research were evaluated using the SPSS 25.0 package program. The measurement data were expressed as mean and standard deviation using independent samples *t*-test or one-way ANOVA; the count data were described by frequency, composition ratio (%), and percentage (%). Pearson correlation analysis was used to explore the relationship between knowledge, attitude, and practice scores. Multiple linear regression analysis was used to analyse the factors influencing ICU nurses' knowledge-attitude-practice towards tracheal intubation patients with PED. In all analyses, *p* < 0.05 was set for significance.

### 3.7. Ethics Review

The study was reviewed and approved by the Institutional Review Board of Lanzhou University (no: LZUHLXY20200030). Informed consent was obtained from all participants.

## 4. Results

### 4.1. Demographic Characteristics

A total of 647 questionnaires were distributed and 627 valid questionnaires were finally returned, with a valid return rate of 96.91%. As shown in Table 1, 79% of the participants were female (*n* = 496), 37% of the nurses were between 26 and 30 years of age (*n* = 233), 69% were unmarried (*n* = 435), and 60% had a Bachelor's degree as their highest level of education (*n* = 379). 53% (*n* = 332) and 47% (*n* = 295) of the nurses were working in the general ICU and specialized ICU, respectively. In addition, 57% of the nurses had worked for 1–5 years (*n* = 359), 63% of the nurses had a junior title (*n* = 393), 20% of the participants were managers (*n* = 123), 19% of the nurses were very satisfied with their job (*n* = 118), 4% were very dissatisfied (*n* = 25), 56% of the nurses were contractual workers (*n* = 354), and 41% of the nurses work 5–8 night shifts a month (*n* = 260) and only 12% do not have to work night shifts (*n* = 75).

### 4.2. ICU Nurses' Knowledge-Attitude-Practice Level of PED

The results in Tables 2 and 3 show that the total score of knowledge-attitude-practice for PED was (18.88 ± 5.18), which was converted to a standard score of (62.90 ± 17.27). The scores of the three dimensions of knowledge, attitude, and practice were (6.46 ± 3.09), (7.53 ± 1.69), and (4.89 ± 2.00), which were converted to a standard score of (64.6 ± 30.90), (75.30 ± 16.90), and (48.90 ± 20.00), respectively. Of the 3 dimensions and the total scores, the attitude dimension scores were good at the upper middle level, the knowledge dimension scores and the total scores of knowledge-attitude-practice were at the lower middle level, and the practice dimension scores were only at the poor level.

### 4.3. Perceived Levels of Each Dimension of the Questionnaire

In the knowledge dimension, nurses had the least knowledge about the incidence of PED (K2, 43%) and the most knowledge about clinical manifestations (K4, 79%). In the attitude dimension, the lowest score is the enthusiasm of learning PED knowledge. In the practice dimension, the scores of those who received PED related training were the lowest, and the scores of those who responded to the department in time when they encountered related problems were the highest. The score range of attitude dimension is 0.71–0.77, and the score range of practice dimension is 0.42–0.55. The results are shown in Tables 4–6.

### 4.4. Univariate Analysis of the Scores of Knowledge, Attitude, and Practice and Total Scores against Sociodemographic and Professional Variables

Statistical differences in the knowledge and attitude scores and total scores were observed in different groups of gender, age, nationality, education, years of work, job satisfaction, and mode of employment (*p* < 0.05). The difference between knowledge score and number of night shifts was statistically significant (*p* = 0.047). The differences between the total score of questionnaire (*p* = 0.008) and attitude scores (*p* = 0.003) and professional title were statistically significant. There were statistically significant differences in attitude scores between their marital (*p* = 0.037) and section (*p* < 0.001). There were statistically significant differences between practice score and nationality, education, professional title, and job satisfaction (*p* < 0.05). More detailed information is given in Table 7.

### 4.5. Multiple Linear Regression Analysis

Multiple linear regression analysis showed that the factors affecting the total score of PED among ICU nurses were age (31–35: *β* = 0.106, *p* = 0.018; 36–40: *β* = 0.199, *p* < 0.001; >40: *β* = 0.203, *p* < 0.001), nationality (*β* = −0.128, *p* < 0.001), professional title (intermediate: *β* = −0.126, *p* = 0.003; junior: *β* = −0.220, *p* < 0.001), job satisfaction (more satisfied: *β* = −0.235, *p* < 0.001; fair: *β* = −0.320, *p* < 0.001; not very satisfied: *β* = −0.261, *p* < 0.001; very dissatisfied: *β* = −0.259, *p* < 0.001), and mode of employment (*β* = −0.110, *p* = 0.009). Gender (*β* = 0.088, *p* = 0.025), age (31–35: *β* = 0.104, *p* = 0.028; 36–40: *β* = 0.177, *p* < 0.001; >40: *β* = 0.169, *p* < 0.001), nationality (*β* = −0.150, *p* < 0.001), and job satisfaction (more satisfied: *β* = −0.122, *p* = 0.018; fair: *β* = −0.220, *p* < 0.001; not very satisfied: *β* = −0.153, *p* < 0.001; very dissatisfied: *β* = −0.205, *p* < 0.001) were the factors that influenced the score of knowledge. The influencing factors of attitude score include gender (*β* = 0.078, *p* = 0.024), age (31–35: *β* = 0.210, *p* < 0.001; 36–40: *β* = 0.305, *p* < 0.001; >40: *β* = 0.369, *p* < 0.001), nationality (*β* = −0.237, *p* < 0.001), section (*β* = −0.088, *p* = 0.007), professional title (intermediate: *β* = −0.208, *p* < 0.001; junior: *β* = −0.332, *p* < 0.001), job satisfaction (more satisfied: *β* = −0.219, *p* < 0.001; fair: *β* = −0.247, *p* < 0.001; not very satisfied: *β* = −0.263, *p* < 0.001; very dissatisfied: *β* = −0.268, *p* < 0.001), and mode of employment (*β* = −0.162, *p* < 0.001). The influencing factors of the score of practice include professional title (*β* = −0.107, *p* = 0.007) and job satisfaction (more satisfied: *β* = −0.202, *p* < 0.001; fair: *β* = −0.261, *p* < 0.001; not very satisfied: *β* = −0.244, *p* < 0.001; very dissatisfied: *β* = −0.159, *p* < 0.001). More detailed information is given in Table 8.

## 5. Discussion

### 5.1. ICU Nurses Need to Improve Their Knowledge, Attitude, and Practice of PEDs

A survey of 627 ICU nurses in some Grade A Hospitals in Gansu Province of China showed that ICU nurses' knowledge, attitude, and practice about PEDs are poor and need to be further improved, especially to regulate their practices.

According to the descriptive statistical analysis, in the knowledge dimension, ICU nurses still do not know the incidence of PED, which may be related to the unclear status of PED research because they do not consult relevant literature, but they have a good grasp of basic knowledge. At the same time, the scores of ICU nurses in the items related to PED bedside assessment methods, surgical methods, and rehabilitation training were relatively low, reflecting that ICU nurses should focus on improving their mastery of PED special expertise. In the attitude dimension, the subjective initiative of ICU nurses to PED independent learning is poor, so it is suggested to take measures such as reward and punishment to promote the initiative of ICU nurses to improve independent learning. In the practice dimension, the frequency of ICU nurses receiving relevant training is low, and the frequency of their feedback-related problems is relatively high, which suggests that the department managers should increase the professional knowledge and skills training about PED and actively take effective measures to solve the PED problems in the nursing process.

### 5.2. Factors Influencing ICU Nurses' Knowledge, Attitude, and Practice about PEDs Are Diverse

The higher the age, the higher the knowledge-attitude-practice score. From the univariate analysis, it was found that ICU nurses aged ≥31 years scored higher than those aged between 20 and 30 years, but as the overall score of knowledge-attitude-practice was at a moderate to low level, it was suggested that nursing managers should give more learning opportunities and training guidance to ICU nurses aged ≥31 years, so that they can give full play to their leading role and guide and supervise young nurses to actively learn, thus creating a good learning atmosphere in the department. This will help to improve their knowledge of PEDs and improve their attitudes. The results of the multivariate analysis found that ICU nurses aged 26–30 years old had no significance in the above scores compared to ICU nurses aged 20–25 years old, which may be related to the smaller age gap and shorter working years. As the ICU is a highly specialized unit, the older ICU nurses had more clinical experience and were better able to anticipate the clinical implications of PED-related treatment and care for patients with extubation than the younger nurses, so they were able to pay some attention to it [30].

The lower the professional title, the higher the knowledge-attitude-practice score. Caring for patients with tracheal intubation and preventing them from developing PEDs is a more specific clinical task, and as most of those with senior and intermediate titles are head nurses or above in management, they have some critical thinking and decision-making power and are responsible for taking control of the whole, but those with junior titles are more focused on specific clinical tasks in the ICU and have more exposure to PED-related knowledge and clinical practice in their work [31]. Those with senior titles scored lower on the attitude dimension, indicating that they place less emphasis on PEDs.

The higher the level of job satisfaction, the higher the total score and each dimension of knowledge-attitude-practice. Studies have shown that nurses' job satisfaction is related to their physical and mental health status [32, 33]. If they are in a long-term physical and mental subhealthy state, it will not only lead to a decrease in job satisfaction, cause a high turnover rate, and reduce work efficiency but also lead to adverse events and reduce their professional identity of nursing work, thus affecting their work attitude. Unit or hospital managers can improve the job satisfaction of ICU nurses by adopting measures such as creating a supportive nursing work environment, developing a reasonable performance allocation system, and providing appropriate incentives, which will increase motivation and help to urge them to learn about PEDs and improve their attitudes and practices.

Formal ICU nurses scored higher than those employed by other forms of employment. Compared to nurses in other modes of employment, they are likely to have more opportunities for further study and training and are therefore better able to acquire more PED-related knowledge, have a more positive attitude, apply their knowledge in their daily nursing work, and have a stronger sense of professional identity.

## 6. Conclusion

This study investigated for the first time the current situation of PED knowledge-attitude-practice of ICU nurses in some tertiary level A hospitals in Gansu Province, analysed the factors affecting their knowledge-attitude-practice level, and, based on the results of the study, proposed methods for improvement, providing a basis and suggestions for better care of PED patients and enabling them to carry out appropriate PED training, which has certain theoretical and practical application value. However, only ICU nurses in some tertiary hospitals in Gansu Province were surveyed, and the location and hospital level of the selected sample had certain limitations, leading to possible regional bias and selection bias. Therefore, the selection of representative hospitals of different grades across the country to expand the sample size is a future research work.

## 7. Implications for Nursing Management

The results of the study showed that the knowledge, attitude, and practice of ICU nurses towards tracheal intubation patients' postextubation dysphagia were in the lower middle level. Therefore, it is necessary to improve the knowledge, attitude, and practice of ICU nurses towards tracheal intubation patients' postextubation dysphagia. This may include, but is not limited to, the development of tools for assessing PED, systematic and professional training, and the development of multidisciplinary collaborative models.

## Figures and Tables

**Table 1:** Demographic characteristics of nurses (*N* = 627).

Characteristics	*N*	%
Gender		
Male	131	20.89
Female	496	79.11
Age (years)		
20–25	137	21.85
26–30	233	37.16
31–35	120	19.14
36–40	78	12.44
>40	59	9.41
Nationality		
The Han nationality	578	92.19
Other nationalities	49	7.81
Marital		
Unmarried	192	30.62
Married	435	69.38
Education		
Health vocational high school	193	30.78
Bachelor's degree	379	60.45
Master's degree and above	55	8.77
Section		
General ICU	332	52.95
Specialized ICU	295	47.05
Years of working in the ICU		
1–5	359	57.26
6–10	181	28.87
11–15	54	8.61
>15	33	5.26
Professional title		
Senior	393	62.68
Intermediate	176	28.07
Junior	58	9.25
Be a manager or not		
Yes	123	19.62
No	504	80.38
Job satisfaction		
Very satisfied	118	18.82
More satisfied	255	40.67
Fair	181	28.87
Not very satisfied	48	7.66
Very dissatisfied	25	3.99
Mode of employment		
Formal	183	29.19
Contract	354	56.46
Personnel agency	90	14.35
Number of night shifts		
0	75	11.96
1–4	146	23.29
5–8	260	41.47
>8	146	23.29

**Table 2:** ICU nurses' scores on KAP dimensions of PED and total scores (*N* = 627).

	Range	Score	Standard score
Knowledge	0∼10	6.46 ± 3.09	64.60 ± 30.90
Attitude	2∼10	7.53 ± 1.69	75.30 ± 16.90
Practice	2∼10	4.89 ± 2.00	48.90 ± 20.00
Total score	4∼30	18.88 ± 5.18	62.90 ± 17.27

**Table 3:** Rating of ICU nurses' total PED knowledge-attitude-practice scores and standard scores for the three dimensions (*N* = 627).

Level	Knowledge, *N* (%)	Attitude, *N* (%)	Practice, *N* (%)
Good	225 (35.89)	190 (30.30)	31 (4.94)
Medium	191 (30.46)	340 (54.23)	179 (28.55)
Poor	211 (33.65)	97 (15.47)	417 (66.51)

**Table 4:** Nursing knowledge about tracheal intubation patients' postextubation dysphagia (PED).

Asked questions	True *N* (%)
K1: definition of postextubation dysphagia (PED)	460 (73.37)
K2: incidence rate of postextubation dysphagia (PED)	269 (42.90)
K3: potential mechanism of PED occurrence	434 (69.22)
K4: clinical manifestation of PED	495 (78.95)
K5: poor outcomes resulting from the PED	474 (75.60)
K6: risk factors for PED	446 (71.13)
K7: assessment method of PED	370 (59.01)
K8: bedside assessment method	352 (56.14)
K9: PED for surgical procedures	355 (56.62)
K10: training methods and therapeutic means to promote the recovery of swallowing function	397 (63.32)

**Table 5:** ICU nurses' attitude towards tracheal intubation patients' postextubation dysphagia.

Subject (*N*, %)	Strongly agree	Agree more	Generally agree	Not agree with	Disagree	Score
A1: interests	172 (27.43)	216 (34.45)	157 (25.04)	43 (6.86)	39 (6.22)	0.74 ± 0.23
A2: initiative study	145 (23.13)	192 (30.62)	195 (31.10)	51 (8.13)	44 (7.02)	0.71 ± 0.23
A3: training	227 (36.20)	199 (31.74)	120 (19.14)	40 (6.38)	41 (6.54)	0.77 ± 0.23
A4: acquisition of knowledge	213 (33.97)	215 (34.29)	116 (18.50)	51 (8.13)	32 (5.10)	0.77 ± 0.23
A5: solve problems proactively	160 (25.52)	217 (34.61)	148 (23.60)	63 (10.05)	39 (6.22)	0.73 ± 0.23
A6: screening and assessment	201 (32.06)	230 (36.68)	113 (18.02)	46 (7.34)	37 (5.90)	0.76 ± 0.23
A7: work procedure	192 (30.62)	230 (36.68)	136 (21.69)	42 (6.70)	27 (4.31)	0.77 ± 0.21
A8: family compatibility	206 (32.85)	223 (35.57)	116 (18.50)	46 (7.34)	36 (5.74)	0.76 ± 0.23
A9: patient functional training	196 (31.26)	233 (37.16)	116 (18.50)	49 (7.81)	33 (5.26)	0.76 ± 0.22
A10: management of PED	199 (31.74)	221 (35.25)	114 (18.18)	63 (10.05)	30 (4.78)	0.76 ± 0.23

A7 and A10 are reverse questions, which have been normalised.

**Table 6:** ICU nurses' practices towards tracheal intubation patients' postextubation dysphagia.

Subject (N, %)	Always	Often	Sometimes	Occasionally	Never	Score
P1: initiative study	31 (4.94)	43 (6.86)	146 (23.29)	240 (38.28)	167 (26.63)	0.45 ± 0.22
P2: receive training	28 (4.47)	40 (6.38)	133 (21.21)	182 (29.03)	244 (38.92)	0.42 ± 0.22
P3: take preventive measures	39 (6.22)	66 (10.53)	185 (29.51)	183 (29.19)	154 (24.56)	0.49 ± 0.23
P4: proactive screening or assessment	32 (5.10)	60 (9.57)	152 (24.24)	189 (30.14)	194 (30.94)	0.46 ± 0.23
P5: adjust your diet	37 (5.90)	79 (12.60)	153 (24.40)	186 (29.67)	172 (27.43)	0.48 ± 0.24
P6: take treatment	41 (6.54)	89 (14.19)	172 (27.43)	182 (29.03)	143 (22.81)	0.51 ± 0.24
P7: exercise to promote the recovery of swallowing function	38 (6.06)	75 (11.96)	155 (24.72)	193 (30.78)	166 (26.48)	0.48 ± 0.23
P8: encourage patients to participate in rehabilitation training	50 (7.97)	106 (16.91)	168 (26.79)	177 (28.23)	126 (20.10)	0.53 ± 0.24
P9: understand the patient's mental state	51 (8.13)	110 (17.54)	157 (25.04)	182 (29.03)	127 (20.26)	0.53 ± 0.24
P10: feedback related issues proactively	102 (16.27)	77 (12.28)	155 (24.72)	151 (24.08)	142 (22.65)	0.55 ± 0.27

**Table 7:** Univariate analysis of knowledge, attitude, and practice and total scores against sociodemographic and professional variables.

	Knowledge		Attitude		Practice		Total scores	
	*t/F*	*p*	*t/F*	*p*	*t/F*	*p*	*t/F*	*p*
Gender	−2.896	0.004	−3.642	<0.001	0.812	0.284	−3.236	0.001
Age	10.134^a^	<0.001	17.081^a^	<0.001	0.830	0.507	10.002^a^	<0.001
Nationality	7.063	<0.001	11.485	<0.001	4.728	<0.001	8.416	<0.001
Marital	−0.527	0.599	−2.085	0.037	0.642	0.521	−0.745	0.457
Education	3.029	0.049	13.260^a^	<0.001	3.638^a^	0.029	5.826^a^	0.004
Section	0.654	0.514	3.773	<0.001	−0.064	0.949	−0.289	0.773
Years of work	7.997^a^	<0.001	10.416^a^	<0.001	0.244	0.865	6.389^a^	<0.001
Professional title	2.204^a^	0.114	6.111^a^	0.003	4.800^a^	0.009	5.032^a^	0.008
Be a manager or not	1.273	0.203	−0.150	0.865	−0.732	0.465	0.387	0.699
Job satisfaction	12.752	<0.001	19.372^a^	<0.001	11.460^a^	<0.001	20.469^a^	<0.001
Mode of employment	7.343	0.001	11.212^a^	<0.001	1.557	0.212	7.858	0.001
Number of night shifts	2.665	0.047	0.353^a^	0.763	1.032	0.378	0.698	0.553

^a^ *p* value is calculated using Welch's *t*-test.

**Table 8:** Multiple linear regression for variables contributing to knowledge, attitude, and practice and total scores.

Variables	B	SE	*β*	*t*	*p*	95.0% CI	
*Total score*							
Constants	25.926	1.099		23.598	<0.001	23.768	28.083
Age							
20∼25	Ref						
26∼30	0.121	0.485	0.011	0.249	0.804	−0.832	1.073
31∼35	1.398	0.591	0.106	2.368	0.018	0.239	2.558
36∼40	3.129	0.721	0.199	4.339	<0.001	1.713	4.546
>40	3.609	0.871	0.203	4.146	<0.001	1.899	5.319
Nationality	−3.515	0.723	−0.128	−4.865	<0.001	−4.934	−2.096
Professional title							
Senior	Ref						
Intermediate	−1.449	0.490	−0.126	−2.955	0.003	−2.412	−0.486
Junior	−3.941	0.759	−0.220	−5.189	<0.001	−5.432	−2.449
Job satisfaction							
Very satisfied	Ref						
More satisfied	−2.476	0.502	−0.235	−4.932	<0.001	−3.462	−1.490
Fair	−3.657	0.529	−0.320	−6.913	<0.001	−4.695	−2.618
Not very satisfied	−5.085	0.771	−0.261	−6.598	<0.001	−6.598	−3.571
Very dissatisfied	−6.855	0.992	−0.259	−6.908	<0.001	−8.804	−4.906
Mode of employment							
Formal establishment	Ref						
Contractual appointment	−0.804	0.489	−0.077	−1.642	0.101	−1.765	0.157
Personnel agency	−1.619	0.615	−0.110	−2.633	0.009	−2.826	−0.412

*Knowledge*							
Constants	7.478	0.888		8.419	<0.001	5.734	9.223
Gender	0.671	0.298	0.088	2.250	0.025	0.085	1.256
Age							
20∼25	Ref						
26∼30	0.331	0.313	0.052	1.056	0.291	−0.284	0.946
31∼35	0.813	0.369	0.104	2.204	0.028	0.089	1.537
36∼40	1.655	0.413	0.177	4.003	<0.001	0.843	2.467
>40	1.783	0.465	0.169	3.836	<0.001	0.870	2.695
Ethnic	−1.723	0.455	−0.150	−3.791	<0.001	−2.616	−0.831
Job satisfaction							
Very satisfied	Ref						
More satisfied	−0.769	0.323	−0.122	−2.381	0.018	−1.403	−0.135
Fair	−1.503	0.340	−0.220	−4.418	<0.001	−2.170	−0.835
Not very satisfied	−1.774	0.493	−0.153	−3.599	<0.001	−2.742	−0.806
Very dissatisfied	−3.229	0.642	−0.205	−5.032	<0.001	−4.489	−1.969

*Attitude*							
Constants	8.272	0.214		38.634	<0.001	7.852	8.693
Gender	0.326	0.144	0.078	2.271	0.024	0.044	0.608
Age							
20∼25	Ref						
26∼30	0.183	0.153	0.052	1.196	0.232	−0.117	0.482
31∼35	0.904	0.183	0.210	4.950	<0.001	0.545	1.263
36∼40	1.564	0.223	0.305	7.027	<0.001	1.127	2.001
>40	2.136	0.267	0.369	8.009	<0.001	1.613	2.660
Ethnic	−1.493	0.224	−0.237	−6.653	<0.001	−1.934	−1.053
Section	−0.063	0.024	−0.088	−2.684	0.007	−0.110	−0.017
Professional title							
Senior	Ref						
Intermediate	−0.782	0.154	−0.208	−5.080	<0.001	−1.084	−0.480
Junior	−1.936	0.234	−0.332	−8.288	<0.001	−2.395	−1.477
Job satisfaction							
Very satisfied	Ref						
More satisfied	−0.753	0.160	−0.219	−4.719	<0.001	−1.066	−0.439
Fair	−0.922	0.168	−0.247	−5.487	<0.001	−1.252	−0.592
Not very satisfied	−1.674	0.245	−0.263	−6.840	<0.001	−2.154	−1.193
Very dissatisfied	−2.318	0.314	−0.268	−7.379	<0.001	−2.935	−1.701
Mode of employment							
Formal establishment	Ref						
Contractual appointment	−0.137	0.155	−0.040	−0.886	0.376	−0.442	0.167
Personnel agency	−0.784	0.194	−0.162	−4.034	<0.001	−1.165	−0.402

*Practice*							
Constants	5.869	0.184		31.917	<0.001	5.508	5.869
Professional title							
Senior	Ref						
Intermediate	−0.147	0.177	−0.033	−0.828	0.408	−0.494	−0.147
Junior	−0.739	0.273	−0.107	−2.710	0.007	−1.275	−0.739
Job satisfaction							
Very satisfied	Ref						
More satisfied	−0.822	0.216	−0.202	−3.803	<0.001	−1.247	−0.822
Fair	−1.155	0.230	−0.261	−5.027	<0.001	−1.607	−1.155
Not very satisfied	−1.835	0.334	−0.244	−5.498	<0.001	−2.491	−1.835
Very dissatisfied	−1.632	0.427	−0.159	−3.821	<0.001	−2.470	−1.632

## Data Availability

All data used to support the findings of this study are included within the article.

## Supplementary Materials

Supplementary Materials
*Instrument*. The Questionnaire on ICU Nurses' Knowledge, Attitude, and Practice of Postextubation Swallowing Disorders in Patients with Tracheal Intubation was the research tool of this study.
