# Supplementary material for: Knowledge, Attitude, and Practice (KAP) of ICU Nurses towards Tracheal Intubation Patients' Postextubation Dysphagia: A Cross-Sectional Study
Source: J Nurs Manag. 2024 May 31;2024:9981458. doi: 10.1155/2024/9981458 (PMC11918944; doi:10.1155/2024/9981458)
Supplement: Supplementary Materials — Instrument. The Questionnaire on ICU Nurses' Knowledge, Attitude, and Practice of Postextubation Swallowing Disorders in Patients with Tracheal Intubation was the research tool of this study. [file 9981458.f1.docx]

The Questionnaire on ICU Nurses' Knowledge, Attitude and Practice of Post-extubation Swallowing Disorders in Patients with Tracheal Intubation

Dear teachers:

Hello everyone! I am conducting a study on “ Knowledge, attitude, and practice(KAP) of ICU nurses towards tracheal intubation patients’ post-extubation dysphagia”. The purpose of this study is to provide a scientific tool. To investigate the current situation of ICU nurses' knowledge-attitude-practice of PED of patients with tracheal intubation, and analyze the influencing factors, so as to provide a basis for nursing managers to develop reasonable, effective and targeted training plans and intervention measures. Thank you very much for completing this questionnaire in your busy schedule. It will take you about 5-10 minutes to complete the questionnaire, and this study will not affect your interests. The data obtained from the survey will be coded and stored securely, and will be published in the form of a group data, and your name and personal data will not be mentioned, nor will the information be disclosed to anyone without your permission. Your name and personal data will not be mentioned and the information will not be shared with anyone without your permission. Please do not consult the relevant knowledge and complete the questionnaire independently. Thank you very much for your support.

a questionnaire on general demographic information of ICU nurses

Please select the option that matches.

1. Gender: □Male □Female
2. Age:  years old.
3. Nationality: □The Han nationality □Other nationalities
4. Marital status: □Unmarried □Married
5. Education status: □Health vocational high school □Bachelor degree □Master and above
6. Section: □Integrated ICU □Specialist ICU
7. Years of working in the ICU: years
8. Professional title: □Senior □Intermediate □Junior
9. Be a manager or not: □Yes □No
10. Job satisfaction: □Very satisfied □More Satisfied □Fair

□Not very satisfied □Very dissatisfied

1. Mode of employment: □Formal □Contract □Personnel agency
2. Frequency of night shifts per month for the last two years: □0 □1-4 □5-8 □>8

The ICU nurses' Knowledge-Attitude-Practice questionnaire on post-extubation dysphagia in patients with tracheal intubation

1 Knowledge

This section was designed to find out the knowledge of ICU nurses related to post-extubation dysphagia. Please select as many options as you think are appropriate. Please do not consult relevant information and complete the questionnaire independently, your cooperation is greatly appreciated.

**K1** Post-extubation dysphagia (PED) is a symptom of post-extubation dysphagia in patients with tracheal intubation who are unable to effectively transfer food from the mouth to the stomach.

□Yes □No □Unclear

**K2** At present, there is no uniform and authoritative data on the incidence of PED at home and abroad, and published studies show that the incidence of PED is relatively high on the whole.

□Yes □No □Unclear

**K3** Potential mechanisms that produce PED include damage to normal anatomical structures, muscle weakness, sensory dysfunction of the mouth, pharynx, and larynx, gastroesophageal reflux, and incoordination of breathing and swallowing.

□Yes □No □Unclear

**K4** The main clinical manifestations of PED include choking on water, no obvious swallowing movement, drool or food coming out of the mouth, changes in eating habits, hoarseness, difficulty or pain in chewing.

□Yes □No □Unclear

**K5** PED is associated with adverse outcomes such as aspiration, aspiration pneumonia, malnutrition, reduced quality of life, increased reintubation rate, ICU length of stay and total hospital stay.

□Yes □No □Unclear

**K6** The main risk factors for PED include the use of sedatives and narcotic drugs in the ICU, tracheal intubation, the presence of tracheal incision, prolonged tracheal intubation and mechanical ventilation, and the number of intubations.

□Yes □No □Unclear

**K7** Because of the disadvantages of the gold-standard test for evaluating PED, which requires special equipment, specialised personnel, a complex procedure and the need to undergo radiation exposure, the bedside evaluation method is still preferred.

□Yes □No □Unclear

**K8** At present, the main methods for bedside assessment of PED in China include drinking water test, standard swallowing function assessment and Gugging swallowing function assessment.

□Yes □No □Unclear

**K9** For patients with severe dysphagia and recurrent aspiration who have failed to respond to rehabilitation or compensation, surgical procedures such as vocal cord endotranslocation, laryngotracheal dissections, cricopharyngeal muscle amputation, nasopharyngeal closure, or gastro/jejunostomy may be used.

□Yes □No □Unclear

**K10** The training and treatment methods to promote the recovery of swallowing function include oral sensory training, oral motor training, low-frequency electrical stimulation, balloon dilatation, and acupuncture therapy.

□Yes □No □Unclear

2 Attitude

This section was designed to find out the status of ICU nurses' attitudes towards knowledge related to post extubation dysphagia. Please select the option that matches. Thanks for your cooperation.

| **Subject** | **Strongly agree** | **Agree more** | **Generally agree** | **Not agree with** | **Disagree** |
| --- | --- | --- | --- | --- | --- |
| **A1** Interested in knowledge about PEDs. |  |  |  |  |  |
| **A2** Ability to take the initiative to learn about PEDs. |  |  |  |  |  |
| **A3** Training on PEDs is necessary. |  |  |  |  |  |
| **A4** Nurses are able to acquire knowledge of PEDs, preventive and management measures. |  |  |  |  |  |
| **A5** When patients have PED problems, you can take the initiative to solve them with your knowledge. |  |  |  |  |  |
| **A6** Screening or assessment of PEDs by healthcare professionals with considerable expertise is an effective measure to prevent their adverse outcomes and is to be done in a teamwork mode. |  |  |  |  |  |
| **A7** There is no need for a well-established process for screening or evaluating PEDs, routine management measures, and documentation of workups. |  |  |  |  |  |
| **A8** Reinforcing the importance of PED for patients and families and improving cooperation is necessary. |  |  |  |  |  |
| **A9** The nurse can guide the patient to exercise the swallowing function properly. |  |  |  |  |  |
| **A10** Enhanced management of PEDs is irrelevant to the treatment and prognosis of a patient's disease. |  |  |  |  |  |

3 Practice

This section was designed to understand the practice of ICU nurses in their daily work with tracheally intubated patients regarding post-extubation dysphagia. Please fill in according to the actual situation. Thanks for your cooperation.

Note: This section surveys the number of times in the last two months.

| **Subject** | **Always**  **( >9 )** | **Often**  **(7-9)** | **Sometimes**  **(4-6)** | **Occasionally**  **(1-3)** | **Never**  **( 0 )** |
| --- | --- | --- | --- | --- | --- |
| **P1** The number of times to actively learn PED related knowledge. |  |  |  |  |  |
| **P2** The number of PED-related trainings received. |  |  |  |  |  |
| **P3** The frequency with which you proactively take appropriate preventive measures to avoid or reduce risk factors and adverse outcomes of PED in your patients. |  |  |  |  |  |
| **P4** The number of proactive screenings or assessments of PED status. |  |  |  |  |  |
| **P5** To ensure dietary safety and prevent malnutrition, you will recommend how often to adjust the patient's diet based on the results of the PED screening or evaluation. |  |  |  |  |  |
| **P6** When patients have adverse outcomes such as aspiration, aspiration pneumonia, or increased reintubation rates due to PED, the frequency with which you take timely and appropriate treatment to alleviate your symptoms. |  |  |  |  |  |
| **P7** The frequency with which you make relevant recommendations and adopt training that promotes the recovery of swallowing function such as oral sensory training techniques, oral motor training techniques or airway protection methods. |  |  |  |  |  |
| **P8** The frequency of proactively encouraging patients to participate in rehabilitative exercises for swallowing function. |  |  |  |  |  |
| **P9** In the process of nursing, in order to timely understand their psychological status, you take the initiative to ask patients and family members how often they feel. |  |  |  |  |  |
| **P10** Proactively provide feedback to the department on the frequency of issues related to PEDs in the nursing process. |  |  |  |  |  |
